# A review of national policies and strategies to improve quality of health care and patient safety: a case study from Lebanon and Jordan

**DOI:** 10.1186/s12913-017-2528-1

**Published:** 2017-08-16

**Authors:** Fadi El-Jardali, Racha Fadlallah

**Affiliations:** 1 Faculty of Health Sciences, Department of Health Management and Policy, American University of Beirut, Riad-El-Solh Beirut, Beirut, 1107 2020 Lebanon; 2 Center for Systematic Review in Health Policy and Systems Research (SPARK), American University of Beirut, Beirut, Lebanon; 3 Department of Clinical Epidemiology and Biostatistics, McMaster University, Hamilton, ON Canada

**Keywords:** Quality improvement, Patient safety, National quality policy, Accreditation, Health system

## Abstract

**Background:**

Improving quality of care and patient safety practices can strengthen health care delivery systems, improve health sector performance, and accelerate attainment of health-related Sustainability Development Goals. Although quality improvement is now prominent on the health policy agendas of governments in low- and middle-income countries (LMICs), including countries of the Eastern Mediterranean Region (EMR), progress to date has not been optimal. The objective of this study is to comprehensively review existing quality improvement and patient safety policies and strategies in two selected countries of the EMR (Lebanon and Jordan) to determine the extent to which these have been institutionalized within existing health systems.

**Methods:**

We used a mixed methods approach that combined documentation review, stakeholder surveys and key informant interviews. Existing quality improvement and patient safety initiatives were assessed across five components of an analytical framework for assessing health care quality and patient safety: health systems context; national policies and legislation; organizations and institutions; methods, techniques and tools; and health care infrastructure and resources.

**Results:**

Both Lebanon and Jordan have made important progress in terms of increased attention to quality and accreditation in national health plans and strategies, licensing requirements for health care professionals and organizations (albeit to varying extents), and investments in health information systems. A key deficiency in both countries is the absence of an explicit national policy for quality improvement and patient safety across the health system. Instead, there is a spread of several (disjointed) pieces of legal measures and national plans leading to fragmentation and lack of clear articulation of responsibilities across the entire continuum of care. Moreover, both countries lack national sets of standardized and applicable quality indicators for performance measurement and benchmarking. Importantly, incentive systems that link contractual agreement, regulations, accreditation, and performance indicators are underutilized in Lebanon and absent in Jordan. At the healthcare organizational level, there is a need to instill a culture of continuous quality improvement and promote professional training in quality improvement and patient safety.

**Conclusion:**

Study findings highlight the importance of aligning policies, organizations, methods, capacities and resources in order to institutionalize quality improvement and patient safety practices in health systems. Gaps and dysfunctions identified can help inform national deliberations and dialogues among key stakeholders in each study country. Findings can also inform future quality improvement efforts in the EMR and beyond, with a particular emphasis on LMICs.

**Electronic supplementary material:**

The online version of this article (doi:10.1186/s12913-017-2528-1) contains supplementary material, which is available to authorized users.

## Background

Since the release of the Institute of Medicine’s “To Err is to Human” and “Crossing the Quality Chasm” reports, considerable attention has been given to improving quality and patient safety in health care settings [1, 2]. Quality improvement can strengthen health care delivery systems, improve health sector performance and accelerate attainment of the health-related Sustainability Development Goals (SDGs) [3, 4]. Quality improvement is now prominent on the health policy agendas of governments in low- and middle-income countries (LMICs) [3, 5].

Different health systems have adopted different strategies to promote quality improvement [6, 7]. An overview of quality improvement strategies in 25 member states of the European Union (EU) found that to some extent, all of these countries have implemented accreditation systems, performance indicators, clinical guidelines, patient safety systems, total quality management (TQMs), and systems for getting patient views. These strategies appeared to have contributed to improved quality and safety of health care, and were most effective when used in combination [8]. Nonetheless, the member states differed in the degree to which national quality improvement policies have been utilized to enhance the effectiveness of approaches to quality improvement. The existence of such policies has been shown to influence the implementation of quality improvement activities in healthcare organizations, especially if they provide information on the quality activities that are needed for an integral system [9, 10].

Another cross-country comparison of quality improvement policies in seven high-income countries (Australia, New Zealand, England, Germany, The Netherlands, Canada and the USA) found that these countries featured national institutes to promote quality improvement activities and report on national quality indicators; however they varied in the degree of government commitment to quality improvement [11].

Given the considerable progress in quality improvement and patient safety initiatives, some EU countries have proceeded to explore the potential for regional harmonization of quality improvement systems and quality indicators [8, 12]. Harmonization refers to the “establishment, recognition and application of common standards and regulatory measures” across countries to minimize inconsistencies, avoid duplication of efforts and facilitate cross-country comparisons [13].

### The EMR context

Member countries of the Eastern Mediterranean Region (EMR) have expressed high commitment to improve quality of care. In 2009, they endorsed a Regional Committee resolution EM/RC56/R.6 on improving hospital performance in the EMR [14]. While some progress has been made in terms of implementing the Patient Safety Friendly Hospital Initiative (PSFHI) across the Region, progress to date has not been optimal. A study of adverse events in 27 hospitals from eight countries, of which six were located in the EMR (Jordan, Tunisia, Egypt, Sudan, Morocco, and Yemen), revealed an average adverse events of 8%, with the range varying between 2.5% and 18% across countries [15]. Similarly, a systematic review of the quality of care in primary healthcare (PHC) in the EMR concluded that the process dimension of quality is an area of major concern [16]. Some of the factors contributing to suboptimal quality and safety in the EMR include the absence of a clear vision and strategic direction to guide and support the implementation of quality and safety interventions, growing role of the private sector, weak public/private collaboration, and absence of institutionalization of quality and safety [14]. Subsequent regional meetings have highlighted the need to undertake baseline assessments of patient safety at hospitals, raise the issue of quality and safety at the policy level and remap the status of health care accreditation in countries of the EMR [5, 17].

The objective of this study was to comprehensively review the national quality improvement and patient safety policies and strategies in two selected countries of the EMR to determine the extent to which these have been institutionalized within the existing health systems. Key lessons will be generated from the study to strengthen the quality and patient safety components of health systems in the selected countries. The findings can also inform future quality improvement efforts in the EMR and beyond, with a particular emphasis on LMICs.

For the purpose of this study, we focused on two LMICs in the EMR, namely Lebanon and Jordan [18, 19], given their similarities in terms of population health outcomes, health system performance and level of health expenditure [14].

### Analytical framework for assessing health care quality and patient safety

We defined quality improvement as the combined and unceasing efforts of all key stakeholders including governments, healthcare professionals, payers, planners, patients, educators and researchers to make the changes that will lead to “better patient outcomes (health), better system performance (care) and better professional development” [20].

Evaluation of a quality system is a complex task, requiring a framework that takes into consideration the multi-dimensional aspects of quality [21]. In 2000, Shaw and Nicholls proposed a framework for evaluating governmental quality initiatives which encompassed four domains: policy, organization, methods, and resources [22]. In 2011, Robert et al. proposed a multi-level framework to study quality, incorporating the macro-level (national health systems), meso-level (health care organizations) and micro-level (clinical teams) [23].

We used a combination of both frameworks to guide the analysis for this study; the framework by Shaw et al. provided the components which we based our analysis on whereas the framework by Robert et al. stratified them into different levels. The adapted framework for this study is presented in Table 1. We purposely focused on the macro- and meso-levels of the health system while acknowledging the interplay across all three levels.

**Table 1:** Analytical framework for assessing health care quality and patient safety

Component	Elements
Macro-level	
Health systems context	• Governance, financial and delivery arrangement of the health system in each country
Policies and legislation	• Presence of an explicit and comprehensive national policy for quality and patient safety • Incentives and disincentives for participation in quality improvement and patient safety initiatives
Organizations & institutions	• Coordination of quality improvement and patient safety initiatives • Accountability and mechanisms to implement and follow up on quality improvement and patient safety initiatives • Support structures for quality: - National council on clinical governance - National society for quality in healthcare
Methods, techniques & tools	• Licensing of health professionals and healthcare institutions • Systems for adverse drug event reporting • National health care accreditation programs • National performance indicators
Meso-level	
Health care infrastructure and resources	• Infrastructure for quality improvement and patient safety • Human resources for health • Health information system • Financial resources

We compared and contrasted the existing quality improvement and patient safety initiatives in the two selected countries across five macro and meso-level components. For each component, we assessed the extent to which the desirable elements were present, the degree of variations within each country, and the degree of variations between the two countries.

## Methods

### Study design

**Fig. 1:**
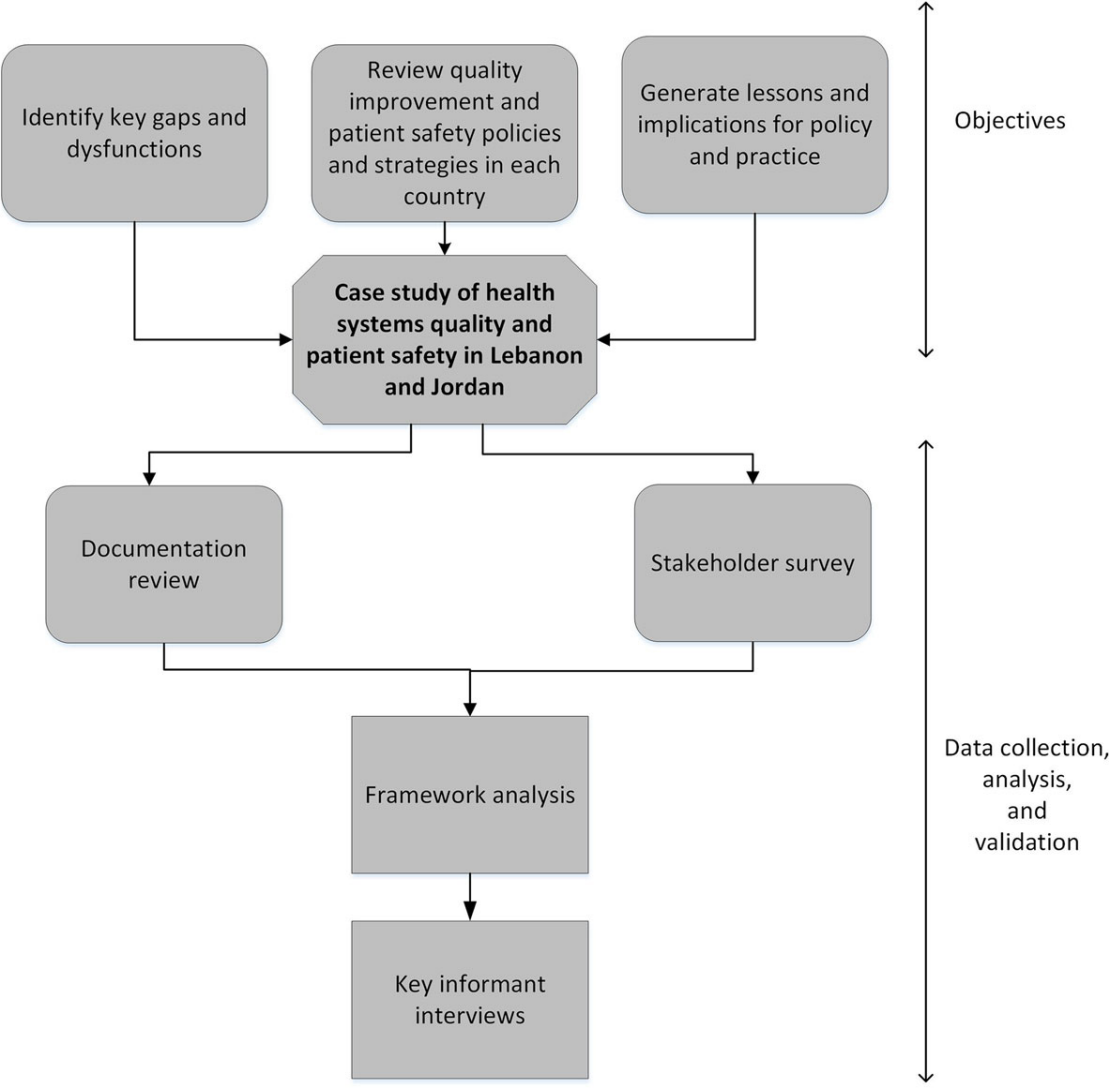
Summary of research activities

The study utilized a mix of quantitative and qualitative research design using a case study approach, and was conducted in two phases (Fig. 1). In the first phase, data was collected in a stepwise approach using documentation review and stakeholder surveys. In the second phase, key informant interviews were conducted to validate findings from the first phase and gain additional insights and feedback. The study extended from March 2015 to June 2015.

### Data collection

#### Documentation review

This step involved a review of research papers, published reports, policy documents and key legislative acts in order to collect data on the health systems context and the existing quality improvement and patient safety policies and initiatives in each country. Documents were identified and obtained from a systematic search of the literature in addition to key stakeholders.

We used Medline and PubMed databases to search for published literature on quality, patient safety and accreditation in Lebanon and Jordan. The search combined various terms for quality and patient safety and included both free text-words and controlled vocabulary terms (see Additional file 1). The electronic database search yielded 231 articles for Lebanon and 226 articles for Jordan, of which 23 and 11 were respectively considered relevant for this study. In addition, we searched the websites of governmental entities and professional bodies including the Order of Physicians, the Order of Nurses and the Syndicate of Hospitals in each country. We also searched the websites of the accreditation organizations operating in each country as well as other relevant organizations such as World Health Organization (WHO), the World Bank, and USAID.

Thirteen documents were also obtained from key stakeholders in Lebanon and Jordan (sampling frame for these stakeholders is detailed in the next section). These included national strategic plans and visions for the health care sector, statistical data on health care accreditation, legislations including law articles, national health account, and reports on health care quality.

Each document was reviewed and summarized in a data collection sheet that included title of the document, type, and component (and elements) of the framework it addressed.

#### Stakeholder survey

The survey tool provided a comprehensive analysis of the existing quality improvement and patient safety initiatives in each country and complemented the data obtained from the documentation review. The survey tool was adapted from the quality improvement questionnaires developed by WHO Regional Office for Europe and International Society for Quality (ISQua) on the basis of the quality framework proposed by Shaw and Nicholls [22, 24]. The questionnaire was pilot-tested with a convenient sample of three stakeholders from Lebanon. Minor changes were made to the questionnaire following pilot-testing. The revised tool included the following three parts and is available in English language (see Additional file 2):

- Part I. National quality improvement and patient safety initiatives: This covered the following subsections: national policies and legislations for quality improvement and patient safety; national organizations and institutions for quality improvement and patient safety; and methods, techniques and tools for quality improvement and patient safety. It targeted national quality experts as well as representatives from the ministry of health and other governmental entities involved in health care quality and patient safety.
- Part II. Health care accreditation program: This provided a comprehensive overview of the national healthcare accreditation program operating in a country. It targeted representatives from healthcare accreditation board, accreditation auditors, and hospitals undergoing accreditation.
- Part III. Infrastructure and resources at the level of health care organizations: This provided an overview of the existing infrastructure, human resources, information system and financial resources for quality improvement and patient safety initiatives at the healthcare organizational level. It targeted healthcare directors and managers, professional bodies/organizations (e.g. Syndicate of Hospitals) as well as external and internal quality auditors.

A sampling frame was developed to identify the selection criteria for the survey. The sampling frame encompassed the following diverse groups of stakeholders: (1) representatives from the Ministry of Health; (2) accreditation program directors and surveyors; (3) quality experts; (4); representatives from professional associations; and (5) directors and managers of health care organizations.

We used the sampling frame to locate the stakeholders. Snowballing was also employed to ensure that other stakeholders involved in health systems quality were included. A total of 19 key stakeholders were identified and approached through targeted emails, of which 17 accepted to participate in the survey (8 from Lebanon and 9 from Jordan). These represented the major stakeholders— ministry of health, national accreditation body, quality experts and public and private health care providers— in the field of health care quality and patient safety in each country. In addition to completing the questionnaire, participants were requested to provide any additional document that may be relevant to this study.

#### Key informant interviews

The semi-structured interviews provided an opportunity to validate the findings from the first phase as well as gain additional insights and feedback from the stakeholders. The interviews were informed by the pooled responses of the survey and documentation review, in addition to a list of prompts developed by the research team. We followed up with the 17 participants who completed the questionnaires, of which only two declined to take part in the interviews. The participants (7 from Lebanon and 8 from Jordan) included representatives from the ministry of health, national accreditation body, and health care organizations. Participants validated the information on quality improvement and patient safety initiatives as well as reflected on gaps and implications for strengthening health systems quality. The interviews were conducted by the lead researcher (FEJ) and lasted between 40 and 60 min each. The interviews were not audio-taped; instead, extensive notes were taken.

Interview transcripts were reviewed and coded by one of the researchers (RF) and subsequently validated by the other researcher (FEJ). Findings were coded according to the analytical framework adopted for this study. Emerging themes were compared to those from the questionnaires and documentation review, and information was added or validated where appropriate.

### Data analysis and synthesis

The data generated from the three sources were collated and analyzed in aggregate form and categorized according to the five components of the analytical framework (Table 1); meaning that findings were analyzed according to components of the framework rather than by source of data.

The first stage of data analysis comprised combining data obtained from the documentation review with the stakeholder survey. The questionnaire was not analyzed quantitatively; instead, the data obtained from the sample within each country were analyzed according to each of the five components of our set framework. Responses were not reported by item but rather grouped according to the components within the analytical framework.

Once the findings from the documentation review were collated with those from the questionnaires, the interviews with stakeholders were conducted. The purpose of these interviews was to validate the findings generated from the first step of analysis and obtain additional insights about each component of the analytical framework.

Data triangulation helped provide a more in-depth understanding of the issue and increase the reliability and validity of findings through cross-checking of information across different data sources.

The study was conducted following standard ethical guidelines and protocols. Participation in this study was voluntary. A verbal consent form was emailed to all participants prior to commencing with the study. Participants were reminded of their right to refrain from participating or withdraw from the study at any time without reprisal. The confidentiality and anonymity of responses were ensured at all times. No names or identifiers were linked to any of the findings emerging from the study.

## Results

We present the findings according to the components described in the analytical framework (Table 1). Where applicable, we complemented the findings with direct quotations from stakeholders.

### Component 1: Health systems context

An overview of the health systems in Lebanon and Jordan revealed variations at the governance, financial and delivery arrangement levels (Table 2). These, in turn, affect the decision-making and regulatory capabilities of governments regarding quality and patient safety. In Lebanon, the private sector is the dominant provider and financer of care. The limited governance capacity of the government, as a consequence of the civil war and the complex political system of the country, has led to the rapid growth and expansion of the private sector in a highly unregulated manner [25]. To alleviate the detrimental effects on the health system structure, the Ministry of Public Health (MOPH) has embarked on a series of health sector reforms [26]. In Jordan, the Ministry of Health (MOH) is the largest single provider and financier of health care services. Governance within the MOH in Jordan is highly centralized, while it is highly fragmented and loosely regulated in the private sector.

**Table 2:** An overview of the health systems arrangements in Lebanon and Jordan

Variable	Lebanon	Jordan
Population size	4,822,000	7,274,000
Life expectancies	Females =82; Males =78	Females = 75; Males = 72
Expenditure on health as % of GDP	7.2%	7.2%
% MO(P)H budget out of total government budget	2.7%	6.7%
Per capita total expenditure on health	US $1092	US $761
Major financing entity	Private sector (71%)	Public sector (61.93%)
% of uninsured population	~46%	~25%
Number of hospitals	Private: 135 Public: 30	Private: 59 Public: 44
% of beds in private hospitals	82.4%	34.3%
Number of primary healthcare centers	213	377

References: [53–58]

### Component 2: Policies and legislation

In both Lebanon and Jordan, commitment to quality improvement and patient safety has not been enacted and exemplified by the development and implementation of the required national policies and reforms that filter down to the operational level. Both countries lack explicit national quality improvement and patient safety policies that define the scope of quality, set out the main objectives of governments to assure quality and patient safety across the continuum of care, clarify roles, responsibilities and relationships, and identify incentives and disincentives for participation in quality improvement and patient safety initiatives. In addition, there are no legislative mandates for health care organizations (in both the public and private sector) to implement specific quality improvement systems or report on a national set of standardized performance indicators for benchmarking and quality improvement. As articulated by one stakeholder from Jordan:

*“Our problem is that we approach quality programs in isolation of the broader picture…what is missing is the continuum; the link from national quality policies that trickle down to the operational level” –*Jordan

Table 3 provides an overview of existing laws alluding to quality and patient safety in each country. These only partially cover health care quality and exist in isolation of a broader quality improvement framework.

**Table 3:** Existing laws alluding to quality and patient safety in each country

Country	Existing laws alluding to quality and patient safety
Jordan	In Jordanian law, the Public Health Code includes articles that emphasize the state’s responsibility to provide healthcare, and the Ministry of Health’s responsibility regarding health matters as follows: - Provide illness prevention, curative measures and supervisory services. - Organize and supervise private and public sector health services. - Provide citizens with health insurance within the allotted parameters. - Establish health, educational and training institutions related to the Ministry and to supervise their management once established.
	As stipulated in Jordanian Law 9 (1999), the High Health Council is responsible for drafting health policies and developing strategic plans as well as planning health services to ensure equitable access to and provision of outstanding health services to all population. Other institutions include Jordanian Medical Council, Supreme Council for Population, Jordanian Nursing Council, National Council for Family Affairs, General Organization for Food and Drug Administration and Department of Joint Procurement
Lebanon	The Ministerial Decree 7612, issued by the parliament in 2002, which amends the legislative decree 139/83 (1983), states that “the MOPH has the right to evaluate, classify and accredit hospitals according to their status, field of specialty and range of services provided”. The decree 482/1 (2009) sets a national Committee for Accreditation of Hospitals, chaired by the Director General of Health.

#### Incentives and disincentives

Although there are no any written policies that identify incentives to participate in quality improvement and patient safety initiatives, the MOPH in Lebanon has been utilizing its funding power to influence hospitals’ behaviors to improve quality, particularly in the private sector which accounts for over 80% of health care service delivery. Specifically, the MOPH has linked accreditation status to contracting with private and public hospitals. This means that hospitals that fail accreditation cannot establish a contract with the ministry and provide services to its patients. As stated by one stakeholder:

*“A major incentive [for hospitals] to seek accreditation is to maintain contract with the ministry… indeed, ministry patients constitute a significant source of revenue for hospitals”-*Lebanon

Nonetheless, linking reimbursement solely to accreditation status was found to be unfair since hospitals placed in the same accreditation category were reimbursed at the same level even if they were not homogeneous in terms of performance or complexity and risk of cases they admit [27]. In 2014, the MOPH implemented a new mixed-model hospital contracting model which was based on a combination of factors which may serve as indicators for risk adjustment [27]. A contracting score is now calculated for each hospital using the following formula: 40% Accreditation +10% Patient Satisfaction +35% Case Mix Index +5% Intensive Care Unit proportion + 5% Surgical/Medical proportion + 5% Deduction proportion by MOPH auditing for inappropriate billing [27]. At the level of primary healthcare (PHC), a performance-based contracting system is being developed for PHC centers that pass accreditation.

This is in contrast to the situation in Jordan where contractual agreements with health care organizations are minimal, making it difficult to control the growing private sector which currently accounts for 34% of total hospital beds in Jordan [28]. Importantly, incentive systems that link contractual agreement, regulations, accreditation status, and performance indicators are absent, thus demotivating health care organizations (in both the public and private sector) to engage in quality improvement and patient safety initiatives.

### Component 3: Organizations and institutions

A positive trend has been observed in both countries regarding the incorporation of quality into the health plans and strategies of the respective ministries of health [28, 29].

In Lebanon, the MOPH has taken responsibility for leading quality improvement and patient safety initiatives; however, a quality unit has not been established at the MOPH and a quality directorate has not been assigned to oversee all quality improvement projects. In Jordan, the concept of quality in healthcare is gaining prominence among the different stakeholders and has been incorporated in the Jordanian’s 2025 vision (unpublished report), the High Health Council’s National Strategy for Health Sector for the year 2015–2019 and the MOH’s Strategic Plan for the year 2013–2017 [28]. To further support its work, a quality unit has been established and a quality directorate assigned at the MOH department. However, the lack of a clear line of responsibility among the different entities involved in quality (Table 3) has been highlighted as contributing to inefficiencies and duplication of work.

In both countries, accountability and mechanisms for implementing quality improvement are not clearly defined. Currently, there is no single coordinating structure to assess and systematically follow up on the implementation of quality improvement initiatives by the many stakeholders involved. In addition, both countries lack a single national academic/resource center for the collection and dissemination of comprehensive comparative information on the health system performance. As stated by one participant:

*“Currently* ***,*** *there are different centers disseminating information on health system performance but the data are scattered and are not reported in one national report”-*Jordan

There is also a shortage of support structures for quality. For instance, both countries lack a national council on clinical governance responsible for the development and implementation of evidence-based clinical guidelines, education and training of healthcare providers in quality and patient safety, and performance appraisals. The existence of such a council can help standardize care and reduce variations in quality across health care organizations. With regards to national societies for quality, these are present in both Lebanon (referred to as Lebanese Society for Quality and Safety in Healthcare) and Jordan (referred to as Jordan Society for Quality); however, they need to be strengthened to play an active role in the decision-making process.

### Component 4: Methods, techniques and tools

This section focuses on licensing requirements, adverse event reporting, health care accreditation programs, and performance indicators, respectively.

#### Licensing requirements

In both Lebanon and Jordan, licensing of health professionals takes place once with no obligatory requirements for re-licensing. The latter is exacerbated by the absence of systems for ongoing performance appraisal, making it difficult to assess and ensure the competency of providers over time. Interestingly, the two countries have different licensing requirements for healthcare organizations. In Lebanon, hospitals and primary healthcare centers (in both the private and public sector) need to be licensed to operate, with no requirements for re-licensing. In Jordan, licensing is restricted to private hospitals and recently, private clinics and polyclinic (as per law 74 (2014)). Also, as per the recent Jordanian law 54 (2014), private hospitals are now mandated to apply for re-licensing on a yearly basis. Public health care organizations do not require licensing or relicensing since they are run by the government.

#### Adverse event reporting

In both countries, adverse event reporting is typically confined to the organizational level, often as part of compliance to accreditation standards. There are no requirements to report adverse events to a national centralized system, with no systematic national enquiries into the occurrence of adverse events in health care.

#### Health care accreditation programs

A major accomplishment in both Lebanon and Jordan is the establishment of national accreditation programs for accrediting health care organizations. A detailed examination of the program in each country revealed significant variations (Table 4).

**Table 4:** Overview of the national accreditation program in Lebanon and Jordan

Features of accreditation program	Lebanon	Jordan
Configuration	- Two national accreditation programs, targeting: - Private and public hospitals - Primary healthcare centers (PHC)	- Health Care Accreditation Council (HCAC) accredits health facilities and services along the continuum from primary to tertiary care
Purpose	- A regulatory tool to strengthen the MOPH’s capability to influence quality of care in both the public and the private sector	- To promote and document improvement in the performance of health care services
Role of government	- MOPH is involved in the development and management of the two national accreditation programs	- HCAC is a private national health care accreditation agency that operates independently of the government
Policy/legislation/decree	- Hospital accreditation by the MOPH is authorized by the Ministerial Decree 7612 (2002) with the functions of the accreditation program also defined by Decree 482/1 (2009) - No legal requirements for PHC accreditation	- HCAC and its functions are not articulated in a law or an official decree
Incentives and disincentives	- Accreditation status linked to MOPH contractual arrangements with hospitals - Ongoing plans to develop a new contractual system for PHC centers that are accredited	- Absence of any commercial or regulatory incentives for health care organizations to seek national accreditation
Standards	- Not ISQua-accredited - Structure- and process-oriented - Not updated on a regular basis *There are ongoing plans to revamp the standards based on ISQua-requirements	- Accreditation standards are all ISQua-accredited - Standards are updated regularly (albeit based on international standards updates rather than country health systems updates)
Process	- No standardized tools to measure compliance with accreditation standards - Performance indicators are not mandated and monitored for compliance - Accreditation status is not renewed on a regular basis - No mechanism in place to ensure quality beyond accreditation *There are ongoing plans to strengthen the accreditation process	- Accreditation status is renewed every two years - Mechanisms in place to ensure quality is sustained in healthcare organizations post accreditation: - Midpoint self-assessment and submission of reports - Unannounced surveys by surveyors - Ongoing plans to introduce a mystery client model
Surveyors	- National surveyors/auditors for PHC accreditation are certified by Accreditation Canada - Ongoing plans to develop and train national surveyors for hospital accreditation - No current plans for re-certification of surveyors	- National surveyors are all ISQua- certified - National surveyors undergo recertification every two years

In Lebanon, there are two national accreditation programs; one for hospitals and one for primary healthcare centers. Both programs have been developed and are currently managed by the MOPH. Hospital accreditation is authorized by Ministerial Decree 7612 (2002) and its functions defined by Decree 482/1 (2009) [27, 30]. The hospital accreditation program in Lebanon constitutes part of the health system arrangement by acting as a regulatory tool to strengthen MOPH’s capability to influence quality of care in both the public and the private sector. Nonetheless, the accreditation program does not cover other types of institutions such as clinics and diagnostic and imaging centers. In Jordan, the Health Care Accreditation Council (HCAC) is a private national healthcare accreditation agency that operates independently of the government and accredits health facilities and services along the continuum from primary to tertiary care [31]. A noted finding is the absence of any commercial or regulatory incentives for health care organizations (in both private and public sector) to seek accreditation by HCAC. This, in turn, may pose a challenge on the sustainability of the national accreditation program in Jordan, as stated by one stakeholder:

*“Attaining accreditation is demanding and expensive, and hospitals are discouraged because we do not have incentives in place”-*Jordan

#### Performance indicators

At present, both Lebanon and Jordan lack national sets of standardized and comparable indicators for hospital performance benchmarking and improvement; however, significant progress has been made in both countries which can be leveraged upon in future initiatives (Table 5). Stakeholders from both countries reflected on the importance of developing national performance indicators:

**Table 5:** Summary of progress with developing national performance indicators in Lebanon and Jordan

Country	Progress with developing national performance indicators
Lebanon	- Attempts have been made to develop a national set of standardized hospital indicators for performance benchmarking and reporting: [59] - A set of 21 indicators were pilot-tested and validated in key selected hospitals. - Initiative is currently pending due to political interferences - Initiative can feed into MOPH’s plan to revamp and update the hospital accreditation standards to include key performance indicators and patient safety goals. - At the PHC level, plans are underway to establish a standardized set of national indicators that all PHC centers should report to the MOPH
Jordan	- The Health Care Accreditation Council (HCAC) launched the National Quality and Safety Goals (NQSGs) initiative in February 2009 to develop annual goals related to high-risk areas associated with patient safety: [60] - Healthcare facilities voluntarily commit to the NQSGs. - Facilities that meet the NQSGs within a year receive certificates from the program’s patron.

*“It is critical to have standardized indicators to report on, but data should be generated from within the system so that it won’t be burdensome.”-*Lebanon

*“Currently, there are no national monitoring and evaluation of quality and performance…but this may change as some hospitals will start to establish electronic medical records that have the capabilities to generate indicators”-* Jordan

### Component 5: Health care infrastructure and resources

#### Infrastructure

As stated earlier, healthcare organizations in both countries are not mandated by legislation to implement specific quality improvement and patient safety systems or strategies. Consequently, this has led to variations in the type and extent to which health care organizations in each country have invested in quality improvement and patient safety initiatives. A noted finding is the heavy investment in achieving accreditation status by hospitals, particularly in Lebanon. Much work is needed to ensure that quality and patient safety are institutionalized as part of a culture of continuous quality improvement rather than as a response to fulfilling accreditation standards as is currently the situation. According to stakeholders from both countries:

*“With the exception of a few large hospitals, quality improvement is not integrated in the majority of small- and medium-sized hospitals; it is not part of a culture of continuous quality improvement.”-* Lebanon

*“Quality improvement plans are integrated only in those organizations working towards accreditation”-*Jordan

#### Human resources

Analysis pointed to weaknesses in both countries with respect to training and developing the capacity of the health workforce (including healthcare managers and leaders) on how to implement, follow up and evaluate quality improvement and patient safety initiatives as well as act on the recommendations. As re-iterated by directors of healthcare organizations from both countries, much of the current efforts in building capacity are channeled towards the enforcement of accreditation standards.

A main reported reason for the suboptimal capacity in quality improvement and patient safety in both countries is the absence of explicit healthcare institutional policies requiring training or continuing medical education of providers in quality improvement and patient safety initiatives. Another reason related to the fact that education about quality improvement and patient safety is not systematically incorporated and emphasized in the curricula of medical students and trainees. For example, a study conducted in Lebanon found that 85% of medical students did not receive any course related to quality improvement, and 93% acknowledged the need to be taught such material [32].

Moreover, providing health personnel with protected time to participate in quality improvement and patient safety initiatives has not been systematically applied across healthcare organizations in both countries; consequently, these initiatives end up being perceived as another ‘program’, additional to the routine activity and not as a tool to improve it. Additional challenges common to both countries include shortages in staffing and work overload which negatively affect patient outcomes and safety [33–36].

#### Health information system

Healthcare organizations in both countries typically generate patient-related data for internal purposes; however, there are no standardized data collection forms, measurement tools, or reporting systems, thus resulting in fragmentation of information within each country. As stated by one stakeholder:

*“While most hospitals and PHC centers generate their own specific patient data sets, these are not standardized across hospitals and PHC centers.”-*Lebanon

Nonetheless, both countries are making efforts to unify and standardize data collection, albeit only in the public sector. For instance, in Lebanon, 77 PHC centers have integrated the MOPH’s Health Information System (HIS) which generates demographic statistics as well as data on inventories and IC-10 indicators (but is yet to incorporate patient medical records). In Jordan, the “HAKEEM” program aims to unify electronic medical records across all public hospitals and health centers. Following the successful application of the program in the pilot phase sites, a plan has been prepared for its wide implementation in the coming years [28].

#### Financial resources

In Lebanon, budgeting for quality improvement initiatives varies across healthcare organizations in the private sector, with some allocating agreed budgets in advance and others failing to systematically budget for quality improvement initiatives. In the public sector, political commitment to financing quality and patient safety initiatives is not regularly translated into increased government budgetary amounts. In Jordan, budgeting for quality improvement initiatives is centralized and not based on the individual needs of public facilities. The process is less explicit in the private sector where allocation of budgets for quality is determined by the quality unit or department of each organization.

## Discussion

Findings from this study show that commitment to quality and patient safety requires multi-faceted interventions at multiple levels for improvement efforts to be fully realized and sustained. Both Lebanon and Jordan have made important progress in terms of increased attention to quality and accreditation in their national health plans and strategies, licensing requirements for health care professionals and organizations (albeit to varying extents), investments in health information systems, and in the case of Lebanon, utilization of incentive systems to some degree. However, both countries still lack a coherent program of government policy in these aspects.

A key deficiency highlighted in both Lebanon and Jordan is the absence of an explicit national policy for quality improvement and patient safety across the health system. Instead, there is a spread of several (disjointed) pieces of legal measures and national plans leading to fragmentation and lack of clear articulation of responsibilities across the entire continuum of care. Efforts to provide a strong statutory framework for quality improvement should aim at embedding it into the existing health system funding and provision systems. This would provide the resources required to support capacity improvement in health systems and healthcare organizations as well as ensure mechanisms are in place to harmonize quality improvement and patient safety initiatives at the national level [8, 37].

In both countries, accreditation is increasingly being emphasized in the strategic plans of governments to enhance the capacity of healthcare organizations to provide quality care. While accreditation is a positive indicator that the building blocks are in place to be able to provide quality care, there seems to be an over-reliance on accreditation as the main quality improvement strategy as opposed to promoting thinking about the full spectrum of quality. Beyond accreditation, quality is also about promoting a culture of measurement, transparency and continuous quality improvement, aligning performance assessment to strategic management, and adapting existing strategies and systems to the context [38].

Another noticeable finding in both countries is the absence of national sets of standardized and applicable quality indicators to monitor progress. Quality indicators are increasingly being utilized for performance measurement and benchmarking [10, 39, 40]. In Taiwan, the successful reporting system that required reporting of 139 indicators by Taiwanese hospitals led the Bureau of National Health Insurance to consider using this system as a reimbursement method for hospitals [41]. In Qatar, plans have been developed to mandate a set of key performance indicators (15 indicators for PHC and 25 indicators for hospitals) for both the private and public sector as part of the Health Services Performance Agreement initiative. Several studies have noted improvement in quality and performance of health care organizations following implementation of national performance indicators [40, 42, 43].

Importantly, incentive systems that link contractual agreement, regulations, accreditation, and performance indicators are still underutilized in Lebanon and absent in Jordan. In Lebanon, the MOPH has undertaken a series of healthcare reforms to address the rising healthcare costs and inefficiencies [27, 44]. Despite revising the re-imbursement formula for services provided by contracted private and public hospitals, the new arrangement does not include measures and outcomes that reflect hospitals’ actual performances. The addition of outcome measures such as complications index, mortality index, and cash flow margins can permit monitoring of hospital performance [45]. Also, with the exception of the MOPH in Lebanon, incentive systems are not being utilized by private and other public third party payers for contracting and financial reimbursement. In Jordan, incentives systems are absent and contractual agreements are minimal. To strengthen the regulatory role of the MOH, contractual agreements can be promoted and linked to accreditation status, attainment of the Jordanian National Quality and Safety Goals, compliance with a national set of quality indicators, or a combination of the above. This can also help alleviate the problem of an almost two-tiered system for quality improvement in the public and private sector in Jordan. In fact, linking accreditation status to incentives such as access to public funding, preferential re-imbursement, health insurance benefits, contractual agreements, or designation as a medical travel destination has been shown to be an effective mechanism in both high-income countries and LMICs [46, 47].

Owing to the lack of commonly agreed and standardized quality indicators to monitor progress at the national level, it is difficult to determine the impact of the existing quality improvement and patient safety initiatives in each country on health systems performance and patient outcomes. Still, anecdotal evidence from a number of primary studies, albeit mostly related to accreditation, demonstrated positive results. In Lebanon, health care accreditation was perceived as a worthy investment, with favorable views mostly related to its effect on enhanced quality and patient safety culture and improved patient satisfaction at both hospitals and primary health care centers [48, 49]. Furthermore, health care professionals indicated that accredited hospitals were more easily able to incorporate quality indicator measurements into their daily activities and to reap the support of staff for such initiatives [50]. In Jordan, the national accreditation program was reported to significantly improve patient satisfaction. Both physicians and nurses had positive perceptions of accreditation standards related to management and leadership, strategic planning for quality, human resources utilization, quality management, and accreditation process and implementation [51]. Moreover, compared to non-accredited Jordanian hospitals, return of patients to the intensive care unit within 24 days of discharge and staff turnover have both decreased in accredited hospitals (albeit those accredited by Joint Commission International (JCI)), with subsequent cost saving of US $38,588 and US $33,333, respectively [52].

While beyond the scope of this study, additional insights and feedback were obtained from the semi-structured interviews which are briefly discussed below. First, stakeholders from both countries demonstrated high interest in the quality improvement and patient safety framework and tool adapted for this study as it approaches quality and patient safety from a multi-level perspectives and stimulates thinking about the full spectrum of quality. Second, stakeholders from both countries initiated discussions about the potential for regional harmonization of quality improvement and patient safety initiatives. A key emerging area related to the identification of a common set of indicators for performance benchmarking across both countries. Linking incentives and reimbursement to specific performance indicators could later be introduced once the capacity is built for collecting and reporting evidence-based performance indicators. Nonetheless, there was consensus among stakeholders that regional harmonization should only be considered once harmonization has been achieved at the national level. These findings warrant further elaboration in future studies.

### Limitations of the study

A limitation of this study is the inclusion of only two countries from the region, thus the findings may not be generalizable to all EMR context. It is important to scale up the study to other countries in the region including the Gulf countries. In addition, due to the focus of this study, we could only analyze the macro- and meso-level components. Additional studies are needed to examine the micro-level components and the interplay across all three levels to achieve health care quality and patient safety.

### Implications for policy and practice

The findings and gaps highlighted in this study can act as a basis to inform national deliberations and dialogues among key stakeholders in each study country. They can also inform future quality improvement efforts in the EMR and beyond, with a particular emphasis on LMICs. An additional contribution of our study is the adaptation and refinement of a framework and tool that can be used by other researchers to assess health systems quality improvement and patient safety initiatives.

In order to institutionalize quality improvement and patient safety practices in health systems, it is critical to ensure that policies, organizations, methods, capacities and resources for quality improvement and patient safety are aligned and integrated.

At the macro-level, governments should consider developing an explicit national policy for quality improvement and patient safety; one that sets out the roles of governments and other stakeholders in assuring quality and patient safety across the health system, clarifies responsibilities and relationships, and identifies incentives and non-incentives including consequences of poor performance. As national policies require implementation considerations, it would be critical to have an overarching regulatory framework that would embed quality improvement and patient safety into existing health system funding and provision systems. This could be complemented by accountability and support structures to facilitate implementation and follow up on quality improvement and patient safety initiatives. Considerations could also be given to the establishment of a national council on clinical governance to oversee the development and implementation of evidence-based guidelines, performance appraisals, and education and training of healthcare providers in quality improvement and patient safety.

Equally important is the need to develop programs and strategies to support measurement and evaluation of quality and patient safety. For this purpose, governments could consider establishing a national set of standardized and applicable quality and patient safety indicators. This could be overseen by a national institution (developed through public/private partnerships) for measuring, monitoring and benchmarking of quality and providing guidance and support to hospitals and primary healthcare centers.

At the meso-level, health care organizations should strive to establish a balance between professional self-regulation and external quality control (e.g. accreditation, licensing, and performance indicators) to guarantee the quality of health services expected by consumers and providers. Considerations should also be given to strengthening quality improvement infrastructure and clinical governance within health care organizations. Finally, cultural change would require significant leadership to institute a cultural of continuous quality improvement, promote systems thinking, and ensure long-term commitment to new learning which is essential for any system seeking transformation.

As countries progress towards national harmonization of quality improvement and patient safety initiatives at the national level, they may consider initiating discussions about potential areas for regional harmonization of quality improvement and patient safety initiatives. A starting point could be the identification of a common set of indicators for performance benchmarking and quality improvement across countries.

## Conclusion

Findings from this study highlight the importance of aligning policies, organizations, methods, capacities and resources in order to institutionalize quality improvement and patient safety practices in health systems. Key gaps and dysfunctions identified can be used to inform national deliberations and dialogues among key stakeholders in each study country. Findings can also inform future quality improvement efforts in the EMR and beyond. The methodology and framework developed for this study can be replicated in future case studies to assess health systems quality improvement and patient safety initiatives.

## Additional files

Additional file 1: Search strategy. (PDF 189 kb)

Additional file 2: Survey tool to assess health systems quality improvement and patient safety initiatives. (PDF 411 kb)

## Abbreviations

EMR: Eastern Mediterranean Region
EU: European Union
HCAC: Health Care Accreditation Council
ISQUA: International Society for Quality in Healthcare
JCI: Joint Commission institution
LMIC: low and middle income countries
MOH: Ministry of Health
MOPH: Ministry of Public Health
NQSGs: National Quality and Safety Goals
PHC: Primary healthcare
TQMs: Total quality management programs
WHO: World Health Organization
