# Supplementary material for: A review of national policies and strategies to improve quality of health care and patient safety: a case study from Lebanon and Jordan
Source: BMC Health Serv Res. 2017 Aug 16;17:568. doi: 10.1186/s12913-017-2528-1 (PMC5559834; doi:10.1186/s12913-017-2528-1)
Supplement: Supplementary file 1 — Search strategy. (PDF 189 kb) [file 12913_2017_2528_MOESM1_ESM.pdf]

Additional file 1: Search Strategy

Quality improvement and patient safety initiatives in Lebanon

Database: PubMed

Last searched: April 26, 2015

Search	Query	Items found
#45	Search **#43 AND #32 AND #17**	231
#44	Search **#43 AND #32**	5579
#43	Search **#42 OR #40 OR #37 OR #34 OR #18**	5117428
#42	Search **"Total Quality Management"[Mesh]**	11909
#40	Search **"Benchmarking"[Mesh]**	10477
#38	Search **Benchmarking**	13181
#37	Search **"Patient Safety"[Mesh]**	6640
#35	Search **PATIENT SAFETY**	89352
#34	Search **"Quality Indicators, Health Care"[Mesh]**	13833
#32	Search **#30 OR #31**	15420
#31	Search **LEBANON**	15420
#30	Search **"Lebanon"[Mesh]**	2861

Quality improvement and patient safety initiatives in Jordan

Database: Pubmed

Last search: April 20, 2015

Search	Query	Items found
#20	Search **#17 AND #18 AND #19**	226
#19	Search **#3 OR #5**	23601
#18	Search **#2 OR #7 OR #9**	5115351
#17	Search **#13 OR #16**	240220
#16	Search **"Academic Medical Centers"[Mesh] OR "Hospitals, Teaching"[Mesh]**	72793
#13	Search **"Hospitals"[Mesh]**	209146
#9	Search **"Quality of Health Care"[Mesh]**	5107229
#7	Search **"Quality Improvement"[Mesh]**	7569
#5	Search **"Jordan"[Mesh]**	2743
#3	Search **jordan**	23601
#2	Search **"Accreditation"[Mesh] OR "Joint Commission on Accreditation of Healthcare Organizations"[Mesh]**	16362
