# Supplementary material for: A review of national policies and strategies to improve quality of health care and patient safety: a case study from Lebanon and Jordan
Source: BMC Health Serv Res. 2017 Aug 16;17:568. doi: 10.1186/s12913-017-2528-1 (PMC5559834; doi:10.1186/s12913-017-2528-1)
Supplement: Supplementary file 2 — Survey tool to assess health systems quality improvement and patient safety initiatives. (PDF 411 kb) [file 12913_2017_2528_MOESM2_ESM.pdf]

Additional file 2: Survey tool to assess health systems quality improvement and patient safety initiatives

<u>Part I: National Quality Improvement and Patient Safety Initiatives</u>

1: National policies and legislations for quality improvement and patient safety

**Statement**	**Yes (Y)**	**No (N)**	**Comment (if needed)**
**1.1 Environment and policy context**			
a. There is a national policy/legislation for quality and safety of health care.			
b. There is a statutory legal requirement for health care organizations to implement specific quality improvement strategies/systems.			
c. There is a clear vision and strategic direction to guide and support the implementation of quality and safety interventions in health care organizations.			
d. There is a standardized set of national indicators for health care organization performance benchmarking and improvement.			
e. There is a standardized national definition for quality (e.g. technical, social, economic, etc…).			
**1.2 There is a national policy/legislation that:**			
a. commits the ministry of health to lead quality improvement by example and to ensure that quality remains visible on every management agenda			
b. identifies the responsibilities and accountabilities of public, private and professional bodies (e.g. for training, monitoring)			
**1.3. Incentives and disincentives for quality and patient safety**			
a. The national policy/legislation identifies incentives to motivate staff and health care organizations to participate in quality improvement and patient safety initiatives.			
b. The national policy/legislation identifies consequences for health care organizations that fail to engage in quality improvement activities.			
c. Quality performance is a requirement in the contractual agreement.			

2: National organizations and institutions for quality improvement and patient safety

**Statement**	**Yes (Y)**	**No (N)**	**Comment (if needed)**
**2.1 Coordination of quality improvement is clearly defined**			
a. There is an identified quality unit and a named accountable officer at the Jordanian Ministry of Health.			
b. An organizational chart identifies sections within the ministry which contribute to quality improvement, and shows relationships between them.			
c. A published annual report identifies quality activities and quantified improvements in performance of the health care system.			
**2.2 Accountability and mechanisms for implementing quality improvement**			

a. Quality improvement and patient safety is explicitly incorporated into national health plans/programs.			
b. Implementation of national guidance (e.g. reports, ministry/government advices) is followed up through performance management or independent review in primary, secondary and tertiary services.			
c. The same requirements for health care quality improvement are in place for both public and private health care organizations.			
**2.3 Support structures**			
a. There is a national association/society for quality and patient safety in health care.			
b. There is a national quality committee/organization representing consumers, providers, insurers, and professions.			
c. There is a resource center for the collation and dissemination of comprehensive comparative information on health system performance (quality, quantity, cost, etc…).			

3: Methods, techniques and tools for quality improvement and patient safety

Statement	Yes (Y)	No (N)	Comment (if needed)
**3.1 Statutory mechanisms for quality and patient safety**			
a. There are mandatory public reporting systems with common indicators among all health care organizations to monitor and evaluate the implementation of quality initiatives.			
b. Existing information systems allow quality and performance to be monitored on a continuing basis across the health system.			
c. Doctors, dentists, nurses and allied practitioners are licensed.			
d. Practitioners are periodically re-licensed.			
e. Medical equipment and drugs are licensed.			
f. Private and public health care organizations are licensed.			
g. There are specific minimum quality and safety indicators for licensing of health care organizations.			
h. There are specific minimum quality and safety indicators for licensing of primary healthcare centers.			
i. Health care organizations undergo periodic re-licensing.			
**3.2 Voluntary external quality assessment and improvement programs**			
a. There are systematic, confidential national enquiries into the occurrence of adverse events and outcomes in health care.			
b. There is a national external quality assurance system for clinical laboratories.			
c. There is a national health care accreditation program in your country			
d. Health care accreditation programs are recognized, endorsed and promoted by the national government.			
e. There is a formal mechanism by which voluntary external quality assessments align with and complement statutory/legal investigations and inspections.			

Part II: Health Care Accreditation Program

Statement	Hospitals		PHCs		Comment (if needed)
	Yes (Y)	No (N)	Yes (Y)	No (N)	
**1.1 Policies, values and culture for accreditation**					
- There is a national law or decree requiring accreditation in your country					
- The accrediting organization is formally authorized by national legislation, official decree, or both.					
- The functions of the accreditation program are defined in any national law.					
- The policy and management of the accreditation program remain consistent, regardless of changes in government.					
- Accredited organizations are automatically assumed to be (re)licensed.					
- There is representation on the accrediting body of the major leading health care professional organizations within Jordan					
**1.2 Incentives and disincentives for accreditation**					
- Accreditation is a requirement for the ministry of health to contract with health care organizations.					
- The ministry of health and other government entities reimburse health care organizations, conditional on the attainment of certain performance indicators specified in accreditation program.					
- Public health insurers and other funding agencies provide financial incentives for health care organizations to encourage accreditation.					
- Private health insurers and other funding agencies provide financial incentives for health care organizations to encourage accreditation.					
- There are consequences for health care organizations that fail accreditation.					
**1.3 Program configuration**					
- The purpose of the accreditation program is to promote and document improvement in the performance of health care services.					
- Participation in the accreditation program is voluntary.					
- The same program includes public and private facilities.					
- The accreditation program constitutes a single national program.					
- The accreditation program covers private and public primary, secondary and tertiary care.					
- The accreditation program covers private and public polyclinics					
**1.4 Organization and structure**					
- The accreditation program is governed by a Board which represents professional, public and governmental interest.					
- Statutory licensing and registration of individuals and organizations are clearly differentiated from accreditation.					
**1.5 Accreditation standards**					
a. Accreditation standards are validated/updated on a regular basis					
b. The current standards include and refer to external guidance such as the ISQUA principles for standards					

c. Professional associations, users, government, and the public are involved in defining and assessing standards.					
d. There are specific criteria that are used to update the standards.					
**1.6 Process of accreditation**					
e. Performance indicators are mandated and monitored for compliance.					
f. There is a standardized tool for measuring the performance indicators.					
g. Procedures and results are made transparent and fair.					
h. There are mechanisms in place to ensure quality is maintained and sustained beyond the accreditation period.					
i. There are certified national accreditation surveyors/auditors.					
j. The accreditation surveyors/auditors are trained and re-validated periodically.					
**1.7 Resources for accreditation**					
- There is adequate funding for sustainability of accreditation program.					
- The accreditation program has access to aggregated data which are routinely reported to the ministry of health and other entities by health care providers.					
- Health care organizations provide health personnel with accurate, complete and timely data by which clinical and organizational performance can be measured.					
- Health personnel are trained to evaluate and improve the performance of their own work and of their health care organization.					
- Health personnel receive formal training on how to enforce accreditation standards.					
- Health personnel receive formal training on how to act on quality improvement opportunities based on recommendations from accreditation surveys					
- Directors of health care services have formal training in accreditation requirements.					

Part III: Infrastructure and Resources at the Level of Health Care Organizations

Statements	Yes (Y)	No (N)	Comment (if needed)
**1.1 Infrastructure**			
a. Quality improvement plans and mechanisms are integrated within health care organizations (e.g. clinical governance, continuous quality improvement, staff and patient satisfaction surveys, audit, performance measurement, risk management).			
b. Performance analysis and improvement are included in the continuing professional development program provided by health care organizations.			
**1.2 Human resources for health**			
a. Health care leaders instill a culture of continuous quality improvement and patient safety at healthcare organizations.			
b. Directors of health care services have formal training in health management and quality requirements.			
c. Health personnel have protected time to participate in quality improvement and patient safety initiatives.			
d. Health personnel are trained to evaluate and improve the performance of their own work and that of their healthcare organization.			
e. Health personnel receive formal training on how to enforce accreditation standards.			
f. There are incentives for health personnel to engage in quality improvement.			
g. Relevant techniques of quality improvement are incorporated in the curriculum, teaching and examination of all clinical undergraduates.			
**1.3 Information system**			
a. There is an agreed minimum patient data set that should be collected in a standardized manner by health care organizations.			
b. Patient administration systems are designed to generate indicators, indices and data for clinical and administrative review.			
c. Accurate, complete and timely data are available by which clinical and organizational performance can be measured.			
**1.4 Financial resources**			
a. Health service performance /contractual agreements identify agreed quality targets as well as price and volume of clinical activity.			
b. There is a yearly budget allocated for quality improvement initiatives within healthcare organizations.			
